# *TREML2* Mutation Mediate Alzheimer’s Disease Risk by Altering Neuronal Degeneration

**DOI:** 10.3389/fnins.2019.00455

**Published:** 2019-05-15

**Authors:** Ya-Nan Song, Jie-Qiong Li, Chen-Chen Tan, Hui-Fu Wang, Meng-Shan Tan, Xi-Peng Cao, Jin-Tai Yu, Lan Tan, the Alzheimer’s Disease Neuroimaging Initiative

**Affiliations:** ^1^Department of Neurology, Qingdao Municipal Hospital, Qingdao University, Qingdao, China; ^2^Clinical Research Center, Qingdao Municipal Hospital, Qingdao University, Qingdao, China

**Keywords:** *TREML2*, neurodegeneration, Alzheimer’s disease, CSF biomarkers, Alzheimer’s disease neuroimaging initiative

## Abstract

A coding missense mutation (rs3747742) in triggering receptor expressed on myeloid cell-like 2 (*TREML2*) has been recently proposed as an important protective factor against Alzheimer’s disease (AD). However, the link between *TREML2* and AD pathology remains unclear. Therefore, we explored the association of *TREML2* rs3747742 with cognitive function, neuroimaging biomarkers and cerebrospinal fluid (CSF) biomarkers related to AD, including CSF total-tau (T-tau), phosphor-tau (P-tau), and amyloid-β (Aβ_1-42_). As for cognitive function, related cognitive scores of Clinical Dementia Rating Sum of Boxes (CDRSB), Alzheimer’s Disease Assessment Scale-cognitive section 11 (ADAS-cog 11), Mini-Mental State Examination (MMSE), and Rey Auditory-Verbal Learning Test (RAVLT) were extracted. We used a multiple linear regression model to examine the association of *TREML2* rs3747742 with the baseline variables. Furthermore, we also calculated the change rate of above variables influenced by *TREML2* rs3747742 via applying a mixed-effects model over a 4-year follow-up. In this analysis, a total of 1,306 individuals from the Alzheimer’s Disease Neuroimaging Initiative (ADNI) database were included. Finally, we observed that only in AD patients, but not in normal controls or mild cognitive impairment (MCI) individuals, *TREML2* rs3747742 exhibited a strong association with CSF total-tau levels at baseline (β = -22.1210, *p* = 0.0166) and 4-year follow-up (β = -0.3961, *p* = 0.0115). Furthermore, no associations were found with CSF Aβ_1-42_ levels, P-tau levels, neuroimaging biomarkers and cognitive function neither for baseline variables nor for longitudinal data. Thus, this study indicated that *TREML2* mediated the risk of AD through influencing AD-related neurodegeneration (abnormal T-tau levels) but not P-tau levels and Aβ pathology.

## Introduction

Characterized by intracellular tau neurofibrillary tangles and extracellular amyloid-β (Aβ) plaques, Alzheimer’s disease (AD) is the most prevalent age-dependent dementia, accompanied by deteriorating cognitive function (Hardy and Selkoe, 2002; Jiang et al., 2012). AD has been suggested as a multifactorial disorder, and the role of genetics in AD pathology has been accepted widely (Querfurth and LaFerla, 2010; Jiang et al., 2012; Karch et al., 2014). Moreover, many biomarkers such as cerebrospinal fluid (CSF) Aβ_1-42_ and tau proteins have emerged as intermediate phenotype approaches in genetic analyses of risk for us to explore the association between genetic variation and process of AD. In the past few years, many genetic variants associated with late-onset AD, such as *APOE*, *BIN1*, *PICALM*, *PLD3*, and *NME8*, have been identified to affect the process of AD pathology via modifying CSF Aβ_1-42_ and tau levels (Schjeide et al., 2011; Liu et al., 2014; Wang et al., 2015; Liu et al., 2016; Wang et al., 2016).

A missense mutation (rs3747742-C) in *TREML2* exhibited the highest linkage disequilibrium (LD) with rs9381040, which is an inter-genic SNP between *TREM2* and *TREML2* showing association with AD risk in the International Genomics of Alzheimer’s Project (Lambert et al., 2013; Benitez et al., 2014). In the previous studies, *TREML2* rs3747742 was identified as a protective factor against AD in Caucasians (Benitez et al., 2014), which was also verified by Jiang et al. in Han Chinese population (Jiang et al., 2017). However, little is known about the mechanism by which this genetic mutation modifies the risk of AD. This study is the first one designed to analyze the role of *TREML2* rs3747742 in the pathogenesis of AD by examining the relation of rs3747742 with CSF proteins, neuroimaging biomarkers and cognitive function in the Alzheimer’s Disease Neuroimaging Initiative (ADNI) database.

## Materials and Methods

### ADNI Database and Subjects

Subjects included in our study were obtained from the ADNI database^1^, which was launched in 2003 as a longitudinal study and recruited participants from almost 63 sites across the United States and Canada (Mueller et al., 2005). Detailed clinical information on the ADNI cohort has been reported previously (Petersen et al., 2010). Here, we restricted our ADNI cohort to general participants (*N* = 1,306), cognitively normal (CN) (*N* = 374), mild cognitive impairment (MCI) (*N* = 705), and AD subjects (*N* = 227) who underwent CSF protein examinations, neuroimaging biomarker measurements and cognitive function tests. Informed consent according to the Declaration of Helsinki was signed by all participants or their authorized representatives.

### Genetic Data

The Illumina Human 610-Quad Bead Chip (including 620,901 SNP and CNV markers) and Illumina Human Omini Express Bead Chip (including 730,525 SNP and CNV markers) were applied for genotyping of ADNI subjects (Saykin et al., 2010). Then these genotype data was made available for ADNI website via sample verification and quality control bioinformatics. Here, we obtained the genotype data of TREML2 (rs3747742) (*N* = 1,306) from the ADNI database. The data for our study were extracted from the ADNI database.

### CSF Biomarker Data

Data for CSF biomarkers, including CSF Aβ_1-42_, total tau (T-tau), and phosphor-tau (P-tau), was obtained from the ADNI database. The acquisition and measurement of CSF data have been described previously (Olsson et al., 2005). These CSF proteins were examined using the xMAP Luminex platform with Innogenetics/Fujirebio AlzBio3 immunoassay kits.

### Neuroimaging Data

The neuroimaging data, including MRI volumes of brain structures, and FDG-PET of cerebral metabolic rate for glucose (CMRgl), was from the ADNI database. The methods for acquisition and processing of cerebral image can be found in prior publications (Desikan et al., 2006). Here, many regions of interest (ROI) analysis, such as brain ventricles, hippocampus, and entorhinal cortex were conducted to calculate their associations with *TREML2* genotypes.

### Neuropsychological Test

To test the influence of *TREML2* rs3747742 on cognitive function, we extracted related cognitive scores of CDRSB, ADAS-cog 11, MMSE, and RAVLT in this study. Finally, the analyses of these baseline cognitive scores and their longitudinal changed scores over 4 years were conducted.

### Statistical Methods

We used Kruskal-Wallis rank sum test to examine the differences in clinical and demographic characteristics of participants included in our analysis. We performed a multiple linear regression model and a mixed-effects model to explore the associations of *TREML2* genotypes with baseline and longitudinal variables, respectively. All above analyses were adjusted for age, gender, education, and *APOE𝜀4* status. Both outcome variables were normalized to z scores to facilitate the comparison between modalities. Statistical significance was considered to have been achieved when *p* < 0.05. Data analyses were performed using R version 3.4.1 statistical software.

## Results

### Demographic Analysis

The demographic characteristics and clinical data of the participants are summarized in Table 1. A total of 1,306 subjects (74.07±7.16 years, 572 women) were recruited. Specifically, 374 CN individuals (74.94 ± 5.53 years, 186 women), 705 MCI patients (73.18 ± 7.59 years, 284 women), and 227 AD patients (75.40 ± 7.72 years, 102 women) were included in this study. As expected, the three groups (CN, MCI, and AD subjects) showed significant differences in MMSE scores and CSF protein levels.

**Table 1:** The characteristics of included subjects at baseline.

Characteristics	All participants (N, mean ± SD)		CN group (N, mean ± SD)		MCI group (N, mean ± SD)		AD group (N, mean ± SD)		*P*-value
Age (years)	1,306	74.07 ± 7.16	374	74.94 ± 5.53	705	73.18 ± 7.59	227	75.40 ± 7.72	<0.001
Gender (male/female)	1,306	734/572	374	188/186	705	421/284	227	125/102	0.011
Education (years)	1,306	15.80 ± 2.91	374	16.28 ± 2.71	705	15.85 ± 2.88	227	14.88 ± 3.08	<0.001
ApoE 𝜀4 (0/1/2)	1,306	702/483/121	374	269/94/11	705	360/276/69	227	73/113/41	<0.001
Genotype (TT/TC+CC)	1,306	648/658	374	182/192	705	353/352	227	113/114	0.899
MMSE (scores)	1,306	27.24 ± 2.57	374	29.08 ± 1.11	705	27.56 ± 1.79	227	23.24 ± 2.05	<0.001
CSF - Aβ (pg/ml)	887	174.95 ± 53.67	252	199.76 ± 52.82	495	171.62 ± 51.81	140	142.06 ± 38.83	<0.001
CSF - T-tau (pg/ml)	880	88.61 ± 51.76	251	69.59 ± 32.38	492	89.23 ± 53.50	137	121.25 ± 55.29	<0.001
CSF - P-tau (pg/ml)	887	37.68 ± 22.00	252	30.40 ± 15.86	495	38.64 ± 22.45	140	47.40 ± 25.26	<0.001


*CN, cognitively normal; MCI, mild cognition impairment; AD, Alzheimer’s disease; MMSE, Mini- Mental State Examination; CSF, cerebrospinal fluid; Aβ, amyloid-β; T-tau, total tau; P-tau, phosphorylated tau. Data are given as mean ± standard deviation unless otherwise indicate. *P*-value indicates the value for the main effect of each subgroup (CN, MCI, and AD group), as assessed with analyses of Kruskal-Wallis rank sum test*.

### *TREML2* rs3747742 and Baseline Variants

**FIGURE 1:**
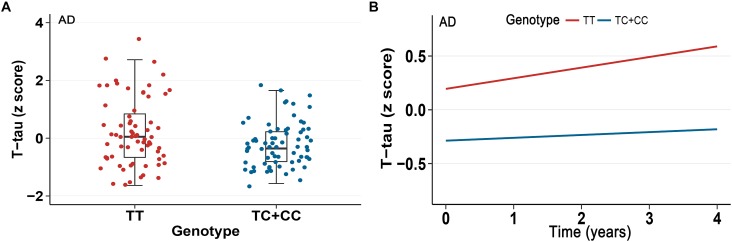
Association between rs3747742 and cerebrospinal fluid (CSF) T-tau levels in AD group. **(A)** Data from multiple linear regression analysis adjusted for age, gender, education, and ApoE𝜀4 status indicated the correlation of rs3747742 with CSF T-tau levels at baseline. **(B)** Data from mixed-effects model adjusted for age, gender, education, and ApoE𝜀4 status indicated the correlation of rs3747742 with longitudinal concentration changes of CSF T-tau during a 4-year follow-up.

In our analysis, we examined the association of a missense mutation (rs3747742) in *TREML2* with CSF proteins, neuroimaging biomarkers and cognitive function among the total participants as well as three clinically diagnosed groups at baseline. Finally, the results indicated that in total participants and AD patients rs3747742 was associated with the level of CSF T-tau, but not with CSF Aβ_1-42_ level, P-tau level, neuroimaging biomarkers and cognitive function (Table 2 and Supplementary Table 1). In total participants, subjects with CC and TC genotypes had lower T-tau levels (β = -7.7764, *p* = 0.0143) when adjusted for age, gender, education, and *APOE𝜀4* status. In addition, AD patients with C allele (CC, TC) had lower T-tau levels than those with TT genotype (β = -22.1210, *p* = 0.0166) when adjusted for age, gender, education, and *APOE𝜀4* status (Figure 1A).

**Table 2:** The correlations of rs3747742 with CSF proteins and cognitive function.

Characteristics		Total		CN		MCI		AD	
		β Coefficient	*P*-value	β Coefficient	*P*-value	β Coefficient	*P*-value	β Coefficient	*P*-value
**Baseline**	CSF-Aβ	2.8717	0.3400	5.0308	0.4147	2.7691	0.4910	-2.4739	0.6817
	CSF-T-tau	-7.7764	0.0143	-4.2753	0.2895	-3.9847	0.3771	-22.1210	0.0166
	CSF-P-tau	-2.1127	0.1330	-0.1286	0.9484	-0.9684	0.6210	-7.7394	0.0653
	CDRSB	0.0085	0.8784	-0.0028	0.8360	-0.0019	0.9770	0.0070	0.9748
	ADAS-cog 11	0.1616	0.5200	-0.3747	0.2083	0.1040	0.7480	1.2402	0.1449
	MMSE	-0.1015	0.2979	-0.3018	0.0074	0.0860	0.5070	-0.2773	0.3111
	RAVLT_immediate	-0.1955	0.6980	0.6234	0.4993	-0.6022	0.3950	-0.7890	0.4426
**Longitudinal**	CSF-Aβ	0.0384	0.4809	0.1045	0.3544	0.0238	0.7540	-0.0964	0.5587
	CSF-T-tau	-0.1548	0.0096	-0.1283	0.2841	-0.0753	0.3523	-0.3961	0.0115
	CSF-P-tau	-0.0949	0.1151	0.0152	0.8960	-0.0500	0.5467	-0.2777	0.0747
	CDRSB	0.0205	0.3503	-0.0860	0.2431	0.0304	0.3432	0.0198	0.7957
	ADAS-cog 11	0.0229	0.4181	-0.0703	0.3471	0.0114	0.7913	0.1200	0.1629
	MMSE	-0.0437	0.0868	-0.1543	0.0175	0.0004	0.9916	-0.0860	0.1950
	RAVLT_immediate	0.0044	0.9039	0.0787	0.3238	-0.0224	0.6966	-0.1084	0.3530


*CN, cognitively normal; MCI, mild cognitive impairment; AD, Alzheimer’s disease; CDRSB, Clinical Dementia Rating Sum of Boxes; ADAS-cog, Alzheimer’s disease Assessment Scale-cognitive section; MMSE, Mini-Mental State Examination; RAVLT, Rey Auditory-Verbal Learning Test; CSF, cerebrospinal fluid; Aβ, amyloid-β; T-tau, total tau; P-tau, phosphorylated tau*.

### *TREML2* rs3747742 and Longitudinal Changes

Furthermore, we also tested the correlations of *TREML2* rs3747742 with longitudinal changes of CSF protein levels, neuroimaging biomarkers and cognition over a 4-year follow-up. We found that total participants (β = -0.1548, *p* = 0.0096) (Table 2) and AD patients (β = -0.3961, *p* = 0.0115) with C allele (CC, TC) showed a slower rate of change in CSF T-tau levels than TT allele carriers after controlling for age, gender, education and *APOE𝜀4* status (Figure 1B), but no associations were found with CSF Aβ_1-42_ levels, CSF P-tau levels, neuroimaging biomarkers and cognitive function (Table 2 and Supplementary Table 1).

## Discussion

This study investigates the association of *TREML2* rs3747742 with CSF protein levels, neuroimaging biomarkers and cognition in total participants as well as CN, MCI and AD subjects. Our main finding was that the coding missense mutation of *TREML2* (rs3747742-C) was closely related to decreased baseline CSF T-tau concentrations and slower rate of longitudinal changes in CSF T-tau levels during the 4 years follow-up in AD patients. Located at *TREML2*, rs3747742 shows the highest LD (*r*^2^= 0.73, *D′* = 0.86) with the GWAS SNP rs9381040 (Benitez et al., 2014), which was confirmed to be the top-significant SNP (*p* = 6.3 × 10^-7^) around *TREML2* (Lambert et al., 2013). It was previously reported that both of the above two minor alleles of rs9381040 (β = -0.02, *p* = 4.11 × 10^-4^) and rs3747742 (β = -0.02, *p* = 1.4 × 10^-4^) were strongly associated with CSF P-tau levels and lower risk of AD (*p* = 1.21 × 10^-5^, CI = 0.88–0.95; *p* = 8.66 × 10^-5^, CI = 0.89–0.96) (Benitez et al., 2014). In the present analysis, we demonstrated rs3747742 was not associated with CSF Aβ_1-42_ level, which was consistent with the result of Benitez et al. (2014). Furthermore, we also detected that rs3747742 was not correlated to CSF P-tau levels but was associated with CSF T-tau levels. This discrepancy may be due to the differences in the grouping of subjects in the two studies. Specifically, the previous analysis was performed in two groups, including the AD group and the control group, but in this study we divided participants into three different clinical subgroups (AD, MCI, and CN groups) (Benitez et al., 2014). Another reason may be the too small sample size of AD subjects in this study, which caused bias in the results of the study. Therefore, more large-scale studies are warranted to confirm this conclusion.

Previous studies have demonstrated that as the functional coding mutation in *TREML2* gene, rs3747742 can encode related proteins and exhibit association with the risk of AD (Cruchaga et al., 2013). TREM2 and TREML2 are structurally similar proteins encoded by the same gene cluster on chromosome 6, which have the opposite effect on the risk of AD. A recent study showed that TREM2 and TREML2 could strictly regulate microglial proliferation, whose dysfunctions may contribute to AD pathogenesis via impairing brain innate immunity (Zheng et al., 2016). TREML2 is a single-pass type I transmembrane protein containing an extracellular Ig-like type V domain and a potential cytoplasmic +xxPxxP SH3-binding motif, which can mediate signal transduction through its cytoplasmic tail (King et al., 2006). Stimulated by inflammatory factors, TREML2 up-regulates the expression of neutrophils and macrophages associated with immune responses, a process that activates immune-related cells to respond to inflammatory stimuli, thereby amplifying the inflammatory response (Klesney-Tait et al., 2006). As for the correlation between neuroinflammation and tau protein, numerous studies shown that proinflammatory cytokines (including IL-1, IL-6, and TNF-α) produced by reactive microglia can induce the pathological modification of tau protein (Li et al., 2003; Quintanilla et al., 2004; Gorlovoy et al., 2009), resulting in neurodegeneration. In addition, it was previously reported that neurodegeneration can influence inflammatory response (Zilka et al., 2009; Stozicka et al., 2010). Also, neuroinflammation mediated by microglia and astrocytes can cause neuronal damage and even death by affecting intracellular mitochondrial function (Wilkins and Swerdlow, 2016). In 2018, the ATN classification system for the diagnostic criteria for AD clearly indicated that CSF T-tau is one of the biomarkers for neuronal damage or neurodegeneration [marked as (N)] (Jack et al., 2016). Furthermore, it also has been speculated that endogenous intracellular tau can be released outside the cells after neurodegeneration (Gomez-Ramos et al., 2006). Soluble extracellular tau can promote neurotoxicity (Gomez-Ramos et al., 2008, 2009) and the release of proinflammatory cytokines, such as IL-1, IL-6, and TNF-α (Kovac et al., 2011). Thus, interactions between tau protein, inflammatory cytokines, and neurodegeneration can lead to the generation of AD pathology. TREML2 will expand the immune-related neuroinflammatory phase to exacerbate this pathological process. Besides, we observed that *TREML2* rs3747742 exhibited a strong association with CSF total tau levels at baseline and 4-year follow up only in AD patients, but not in CN or MCI individuals. We thought the distinct outcomes among three groups about the association between rs3747742 and CSF total tau levels may be due to the disease status. Evidence has shown that disease status may affect the association between genetic variation and gene expression (Rhinn et al., 2013). Therefore, disease status may also affect the association between rs3747742 and CSF total tau levels at baseline and 4-year follow-up. However, the specific mechanism by which *TREML2* rs3747742 affects the expression and function of related proteins with various disease status needs further study and interpretation.

In summary, our findings showed that *TREML2* genetic mutation (rs3747742-C) was associated with CSF T-tau levels in AD patients, suggesting this mutation plays an important role in AD-related neurodegeneration. Neurodegeneration is a common pathway in various neurodegenerative diseases. However, as for other neurodegenerative disease, such as Parkinson’s disease (PD), one study discovered that 24 PD susceptibility variants which identified in GWAS previously were not associated with *TREML2* (Nalls et al., 2014; Chan et al., 2016). Thus, more independent researches with large sample size and diverse ethnicity are required to confirm the role of *TREML2* in AD pathology.

## Author Contributions

J-TY, LT, and Y-NS designed the study, ADNI conducted the subject recruitment and data collection. Y-NS, J-QL, and H-FW analyzed the data. Y-NS, J-TY, C-CT, and M-ST interpreted the findings of study. Y-NS, J-TY, J-QL, and X-PC wrote the manuscript.

## Conflict of Interest Statement

The authors declare that the research was conducted in the absence of any commercial or financial relationships that could be construed as a potential conflict of interest.

## Footnotes

1 http://adni.loni.usc.edu/
