# Supplementary material for: TREML2 Mutation Mediate Alzheimer’s Disease Risk by Altering Neuronal Degeneration
Source: Front Neurosci. 2019 May 15;13:455. doi: 10.3389/fnins.2019.00455 (PMC6529571; doi:10.3389/fnins.2019.00455)
Supplement: Supplementary file 1 [file Table_1.docx]

**Supplementary Table 1.** The correlations of rs3747742 with FDG-PET and structural MRI. Abbreviation: CN, cognitively normal; MCI, mild cognitive impairment; AD, Alzheimer’s disease; CMRgl, Cerebral Metabolic Rate for glucose.

| **Characteristics** | | **Total** | |  | **CN** | |  | **MCI** | |  | **AD** | |
| --- | --- | --- | --- | --- | --- | --- | --- | --- | --- | --- | --- | --- |
|  |  | **β Coefficient** | **P value** |  | **β Coefficient** | **P value** |  | **β Coefficient** | **P value** |  | **β Coefficient** | **P value** |
| **Baseline** | CMRgl | -0.0068 | 0.4137 |  | -0.0039 | 0.7809 |  | -0.0067 | 0.5302 |  | -0.0295 | 0.1690 |
|  | Ventricles | 1.30×10^-3^ | 0.1820 |  | 1.97×10^-3^ | 0.2180 |  | 1.45×10^-3^ | 0.2870 |  | -6.42×10^-2^ | 0.8020 |
|  | Hippocampus | -0.2080 | 0.6979 |  | -6.8300 | 0.9357 |  | -0.5020 | 0.5167 |  | -0.4140 | 0.7577 |
|  | Entorhinal | 1.3500 | 0.9730 |  | 0.3610 | 0.5742 |  | -0.2200 | 0.6973 |  | -0.2660 | 0.7881 |
| **Longitudinal** | CMRgl | 0.0044 | 0.9039 |  | 0.0787 | 0.3238 |  | -0.0224 | 0.6966 |  | -0.1084 | 0.3530 |
|  | Ventricles | 0.0869 | 0.0259 |  | 0.1357 | 0.0699 |  | 0.0617 | 0.2345 |  | 0.0130 | 0.8899 |
|  | Hippocampus | -0.0380 | 0.3534 |  | -0.0649 | 0.4527 |  | -0.0341 | 0.5674 |  | -0.0332 | 0.7692 |
|  | Entorhinal | 0.0242 | 0.5855 |  | 0.0390 | 0.6523 |  | 0.0192 | 0.7685 |  | -0.0155 | 0.8983 |
